# Commentary: Evaluating a digital intervention for symptom management in breast cancer care: insights from oncology nurses

**DOI:** 10.1177/17449871261445358

**Published:** 2026-05-21

**Authors:** Lwandile Tokwe

**Affiliations:** PhD, Faculty of Health Sciences Cape Town, University of Cape Town, Cape Town, South Africa

**Commentary to:** Nurses’ perceptions of a digital supportive intervention for patients with breast cancer receiving neoadjuvant chemotherapy: a qualitative study (Rooth et al., 2026).

The United Nations (Centers for Disease Control and Prevention, 2024) defines chronic diseases as long-term health conditions that persist for a year or more, require ongoing care and account for approximately 41 million deaths annually, representing about 74% of global mortality. Cancer, one of non-communicable diseases (NCDs), affects populations worldwide (Word Health Organization, 2025). The management of NCDs has been facilitated by digital health interventions, which use technology to deliver health services and support knowledge exchange (Murray et al., 2016). Furthermore, these digital health interventions aim to improve the quality of care by capturing and delivering information through a digital platform. This commentary examines the study by Rooth et al. (2026) conducted in oncology clinics in Sweden. It revealed that the Interaktor app can help nurses identify cancer patients needing support and could be integrated into routine oncology practice if usability improvements are made. Nurses also noted that the app promoted patient self-care and symptom communication during treatment, though reporting outside monitoring hours remains a barrier.

## Summary of the paper

This study by Rooth et al. (2026) aimed to describe nurses’ perceptions of an interactive digital intervention (Interaktor) to provide support in symptom management for patients with breast cancer during neoadjuvant chemotherapy (NACT). A qualitative approach using a descriptive design was employed. Data were gathered using paired interviews from four oncology nurse specialists who were purposefully selected in two oncology clinics. Data were analysed using reflexive thematic analysis steps by Braun and Clarke (2021). Two themes were identified (1) taking on a change: use and utility in daily routines and (2) insights for supportive care: the value of integrating a digital intervention. These findings underscore the significance of establishing routines, allocating time and customising user-friendliness. Furthermore, the participants were enabled to identify patients’ needs for additional support and enhance their ability to involve patients in their care. In essence, this study offers valuable insights from the oncology trained nurses on the use of the Interaktor, which is an interactive digital intervention during NACT for patients with breast cancer.

## Relevance of a chosen method

A qualitative approach was appropriate in this study as it enabled the researchers to gather subjective perspectives from the nurses. Furthermore, this approach enabled data gathering within the nurses’ natural setting, namely the two oncology clinics. Brink et al. (2018) noted that a descriptive design is appropriate when more information is needed about a particular phenomenon. Furthermore, a descriptive design was appropriate, as it allowed exploration of a poorly understood phenomenon. Including two oncology clinics was also suitable, as it ensured participation from nurses working in these settings and enabled them to share their perspectives on the intervention. The authors chose a purposive sampling method which was appropriate as they needed nurses who had a specialty in oncology nursing.

The authors conducted paired interviews with four oncology trained nurses. In line with a qualitative approach, this sample size was appropriate, as the focus was on depth rather than numbers. Using a qualitative descriptive design, the study aimed to capture the nurses’ perspectives, emphasising contextual nuances in oncology clinics rather than producing numerical data or generalisable findings (Ahmed, 2024; Cleland, 2017).

## Critical insights

This study offers important contributions by showing that the Interaktor app has the potential to assist nurses in identifying cancer patients in need of support within oncology clinics. In addition, from the nurses’ perspectives, the digital intervention could be effectively integrated into routine oncology practice, particularly if their recommendations are incorporated to improve user-friendliness. Furthermore, nurses reported that access to health information and advice via the Interaktor app promoted self-care among some patients. Engagement with the app enabled patients to better understand and articulate their symptoms during NACT, particularly using the free-text messaging feature. Although nurses found the Interaktor app acceptable, they identified barriers related to patients reporting symptoms after working hours, which falls outside the app’s stipulated monitoring times.

## Strengths and limitations

A key strength of the study is the use of semi-structured interviews with oncology trained nurses who were directly involved in the intervention, enabling the collection of rich, first-hand, experience-based insights. A limitation of the study is that the paired interviews were conducted 6 months after the intervention, which may have introduced recall bias and affected the accuracy of participants’ accounts.

## Implications for nursing practice and policy

The study’s findings have direct implications for clinical practice and policy development:

○ For nursing practice, the Interaktor app through is features such as alerts has the potential to guide care delivery by the oncology trained nurses, through enabling early identification of patient health concerns and facilitating ongoing, responsive support throughout the treatment trajectory. In addition, this study suggests that integrating the Interaktor app into routine care for patients receiving NACT can enhance nursing support during treatment.
○ For policymakers and hospital leaders, these findings underscore the need to address workplace pressures such as time allocation on the app and workplace integration for nurses.
○ For nursing practice, the app may serve as a source of patient-reported data that clinicians can use to inform regimen adjustments and guide the review of treatment plans for cancer patients.
○ The Interaktor app should be developed in users’ native languages to improve understanding and maximise effectiveness. This will also promote reach.

## Future directions

The findings reflect nurses’ perspectives during the treatment phase of cancer care. Future studies could explore the potential benefits of Interaktor during the follow-up period. Future research could focus on testing the Interaktor app in other regions, particularly in low- and middle-income countries where NCDs are prevalent.

## Conclusion

The findings of this study demonstrated that the Interaktor app used for patients with breast cancer receiving NACT was acceptable to nurses across both oncology clinics. Overall, the use of a qualitative descriptive design by Rooth et al. (2026) was appropriate for describing the nurses’ perspectives on a digital supportive intervention for patients with breast cancer receiving NACT.
